# A multiplex real-time PCR assay targeting virulence and resistance genes in *Salmonella enterica *serotype Typhimurium

**DOI:** 10.1186/1471-2180-11-151

**Published:** 2011-06-27

**Authors:** Marie Bugarel, Sophie A Granier, François-Xavier Weill, Patrick Fach, Anne Brisabois

**Affiliations:** 1 French Agency for Food, Environmental and Occupational Health Safety (ANSES) Laboratory for Food Safety, 23 Avenue du Général de Gaulle, F-94706 Maisons-Alfort Cedex, France; 2 Laboratoire des Bactéries Pathogènes Entériques, Centre National de Référence des Salmonella, Institut Pasteur, 28 rue du Docteur Roux, 75724 Paris Cedex 15, France

## Abstract

**Background:**

Typhimurium is the main serotype of *Salmonella enterica *subsp. *enterica *implicated in food-borne diseases worldwide. This study aimed to detect the prevalence of ten markers combined in a macro-array based on multiplex real-time PCR. We targeted characteristic determinants located on pathogenicity islands (SPI-2 to -5, virulence plasmid *pSLT *and *Salmonella *genomic island 1 (SGI1)) as well as a specific 16S-23S rRNA intergenic spacer sequence of definitive type 104 (DT104). To investigate antimicrobial resistance, the study also targeted the presence of genes involved in sulfonamide (*sul1*) and beta-lactam (*bla*_TEM_) resistance. Finally, the *intI1 *determinant encoding integrase from class 1 integron was also investigated.

**Results:**

A total of 538 unrelated *S*. Typhimurium strains isolated between 1999 and 2009 from various sources, including food animals, food products, human and environmental samples were studied. Based on the combined presence or absence of these markers, we distinguished 34 different genotypes, including three major genotypes encountered in 75% of the studied strains, Although SPI determinants were almost always detected, SGI1, *intI1*, *sul1 *and *bla*_TEM _determinants were found 47%, 52%, 54% and 12% of the time respectively, varying according to isolation source. Low-marker patterns were most often detected in poultry sources whereas full-marker patterns were observed in pig, cattle and human sources.

**Conclusion:**

The GeneDisc^® ^assay developed in this study madeit easier to explore variability within serotype Typhimurium by analyzing ten relevant gene determinants in a large collection of strains. This real-time multiplex method constitutes a valuable tool for strains characterization on epidemiological purposes.

## Background

Non-typhoid salmonellosis is one of the most frequently-reported bacterial foodborne diseases and is a major economic and public health issue worldwide. European data show that *Salmonella *is the second most predominant bacterial pathogen, causing around 132,000 human cases in 2008 [1]. In the United States, *Salmonella *serotypes cause an estimated 1.4 million cases of foodborne disease each year [2]. The primary reservoirs of *Salmonella *are food-producing animals, the three main sources being poultry, cattle and pigs. Of the numerous different serotypes, only a few are frequently isolated from human and animal sources. Serotypes Enteritidis and Typhimurium are the most frequently encountered in human and animal sources. Together, they represent 80% of confirmed human salmonellosis cases in Europe, with a marked decrease in serotype Enteritidis cases but an increase in *S*. Typhimurium cases [1]. Serotype Typhimurium was implicated in 47% of the notified foodborne outbreaks in France in 2008 http://www.invs.sante.fr. Of non-human isolates, this has been the most commonly-reported serotype in the French *Salmonella *network in its 15 years of surveillance. Furthermore, in many countries, definitive phage type 104 (DT104) has increased among serotype Typhimurium in the two past decades. Identifying Typhimurium phage types requires maintaining a phage library and specially trained personnel. There is thus a real need, therefore, to develop alternative molecular approaches for identifying Typhimurium DT104 strains. A DNA sequence unique to the DT104 phage type has already been described (16S-23S intergenic spacer sequence) [3,4]. Molecular analysis using relevant gene markers can improve the surveillance and typing of this well-isolated serotype. Markers selected in this study were especially related to virulence and antimicrobial resistance. *Salmonella *pathogenicity is based on the presence of various mobile elements. Five *Salmonella *pathogenicity islands (SPIs) are known to be involved in the virulence expression and invasivity of *Salmonella *[5]. SPI genes encode various functional proteins implicated in cellular invasion and the interaction between host and bacterial cells, such as the type III secretion system and effector proteins. In this study, the presence of four SPIs was investigated by targeting their gene determinants: the *ssaQ *gene implicated in the secretion system apparatus protein (SPI-2), the *mgtC *gene encoding the intramacrophage survival protein for SPI-3, the *spi4D *gene encoding HLYD family secretion protein for SPI-4 and the *sopB *gene implicated in the translocated effector protein of T3SS for SPI-5. The diversity of the *Salmonella *genome is related to the acquisition of plasmids that confer a selective advantage via antimicrobial resistance and/or virulence expression [6]. The common feature of *Salmonella *virulence plasmid loci is a well-conserved 7.8 kb region that plays a major role in the expression of the virulence phenotype in *Salmonella*. This *spv*-locus may be present in serotype Typhimurium isolates and was tested by targeting the *spvC *gene.

*Salmonella *genomic island SGI1 is a 43 kb integrative mobilizable element that confers multidrug resistance and may also be involved in the increased virulence and invasivity of *Salmonella *Typhimurium DT104 strains. SGI1 has also been described in other serotypes, possibly acquired by horizontal transfer [7]. In this study, the presence of SGI1 was investigated by targeting the left junction in the flanking region of SGI1[8]. SGI1 harbors a cluster of genes containing the complex class 1 integron that encodes multidrug resistance, most often associated with the ACSSuT pentaresistance to amoxicillin (*bla*_PSE-1_), chloramphenicol/florfenicol (*floR*), streptomycin/spectinomycin (*aadA2*), sulfonamide (*sul1*) and tetracycline (*tetG*). The 5' well-conserved region including the *intI1 *determinant that encodes integrase from class 1 integron was targeted, as was the *sul1 *gene that codes for resistance to sulphonamides. Antimicrobial resistance to beta-lactams has also been reported in isolates from human and animal sources (6). Resistance mechanisms such as penicillinase hyperproduction, extended spectrum beta-lactamases (ESBL) or inhibitor-resistant TEM beta-lactamase are encoded by the plasmid-mediated *bla*_TEM _gene. The presence and diffusion of *bla*_TEM _genes are a serious public health issue, and could be responsible of treatment failure.

The aim of this work was to develop a simple, easy-to-use tool for *Salmonella *genotyping based on the detection of genes of significant public health concern. The macroarray-based assay was applied to a large collection of serotype Typhimurium isolates representative of various sources and sampled at different times over a 10-year period.

## Methods

### Principle of the GeneDisc^® ^array

The principle of the GeneDisc^® ^array (GeneSystems, Bruz, France, http://www.genesystems.fr) has been described previously [9]. It is a disposable plastic tray the size of a compact disc. Its rim is engraved with 36 reaction microchambers preloaded with desiccated primers and fluorescence-labeled probes for target detection. The GeneDisc^® ^is divided into six sectors, each linked to six microchambers. A duplex real-time PCR can be performed in each microchamber using reporter dye 6-FAM (490-520 nm) or ROX (580-620 nm). Each GeneDisc^® ^can be used to simultaneously investigate six strains in order to detect 12 markers. The 40-cycle thermal PCR program takes 45 minutes. Results are recorded and can also be followed in real time on the computer screen. GeneSystems' GeneDisc^® ^system has been recently used to genotype verotoxin-producing *Escherichia coli *[10].

### GeneDisc^® ^array developed in this study

The GeneDisc^® ^array was designed to simultaneously detect 10 specific gene targets, together with a negative control and a positive *Salmonella *genus control (*ttrC *gene previously described) [11]. This "STM GeneDisc^®^" array was set up as follows: microwell 1) *intI1 *(6-FAM label) and *sopB *(ROX-label); microwell 2) *bla*_TEM _(FAM) and *ssaQ *(ROX); microwell 3) *spvC *(FAM) and *spi_4D *(ROX), microwell 4) DT104 16S to 23S spacer (FAM) and *mgtC *(ROX); microwell 5) *ttrC *gene (FAM) and *sul1 *(ROX); and microwell 6) SGI1 left junction (FAM) and negative control (ROX). The oligonucleotide primers and gene probes used in the GeneDisc^® ^are given in Table 1. All the oligonucleotides were purchased from Sigma-Aldrich (St. Quentin Fallavier, France). GeneSystems (Bruz, France) was responsible for GeneDisc^® ^spotting and manufacturing. All the gene markers are detected with the GeneDisc^® ^system in less than one hour of operation.

**Table 1:** Primers and probes designed for the GeneDisc^® ^assay

Target sequence	Forward primer, reverse primer and probe sequences (5'-3')	GenBank accession number	Location within sequence
DT104	GGACCTGGCTGAGTTTATTTCG		1370 - 1391
16S-23S	GCATCGGCTGTGAGACCAA*	AF275268	1438 - 1420
spacer^a^	FAM-TGGTTTCTGAAAGCGGAGCTAATGCG-BHQ		1393 - 1418

	TCTGCTGAGCGACAACAGATTT		1498146 - 1498167
*ssaQ*^b^	TGGCACCAGCCTGAATATACAG*	AE006468	1498213 - 1498192
	ROX-TCCTGCCCCTCCTGTGGTAGT -BHQ		1498169 - 1498189

	AAGAGGCCGCGATCTGTTTA*		3964669 - 3964650
*mgtC*^c^	CGAATTTCTTTATAGCCCTGTTCCT	AE006468	3964600 - 3964624
	ROX-AAGGGTTAGGTTCGGTCCCCG-BHQ *		3964648 - 3964628

	CGGCGGACTTACTTTTTGAAA		4482051 - 4482071
*spi4_D*^d^	TGGTCACGGTATTTGGGTAATATTT*	AE006468	4482132 - 4482108
	ROX-CCAAAAGTAAGGACTATGCTGGCCG-BHQ		4482077 - 4482101

	CTTATGAGGGAAAGGGCG*		1179300 - 1179283
*sopB*^e^	ATGCACACTCACCGTGG	AE006468	1179215 - 1179231
	ROX-TTGGGATACCAAGAATATTCATCACGCC-BHQ*		1179275 - 1179248

	AATGAACTACGAAGTGGGCG*		24307 - 24288
*spvC*^f^	TCAAACGATAAAACGGTTCCTC	FN432031	24232 - 24253
	FAM-ATGGTGGCGAAATGCAGAGACAGGC -BHQ*		24285 - 24261

	GGATTTTCTCCAGCTTCTGT		132 - 151
Left junction of SGI1^g^	CTAACCATAAGAGAACTTCC*	AF261825	263 - 244
	FAM-TAAATCTCCTAAATTAAATTAAAACGAAGTAAAACC -BHQ		161 - 197

	TGGGCAGCAGCGAAGTC*		27686 - 27670
*intI1*^h^	TGCGTGGAGACCGAAACC	AF261825	27617 - 27634
	FAM-AGGCATTTCTGTCCTGGCTGGCG-BHQ*		27668 - 27646

	CTGGATCTCAACAGCGG		270 - 286
*bla*_TEM_^i^	CAACACGGGATAATACCGC*	AJ634602	378 - 360
	FAM- AGATCCTTGAGAGTTTTCGCCCCG-BHQ		289 - 312

	TCCTGACCCTGCGCTCTATC		29611 - 29630
*sul1*^j^	TGCGCTGAGTGCATAACCA*	AF261825	29679 - 29661
	ROX-ATTGCTGAGGCGGACTGCAGGC -BHQ		29636 - 29657

FAM = 6-carboxylfluorescein; ROX = carboxy-X-rhodamine; BHQ = Black Hole Quencher. * complementary strand; ^a^:marker of the phage type DT104 located in the 16S-to-23S spacer region of bacterial rRNA genes [4]; ^b^: gene encoding SsaQ; ^c^: gene encoding MgtC; ^d^: gene encoding Spi4_D; ^e^: gene encoding SopB; ^f^: gene encoding SpvC; ^g^: marker of the SGI1 left junction which is composed of the end of the *thdF *gene and the intergenic sequence between *thdF *and *int *genes [8]; ^h^: marker targeting the gene encoding the integrase of the class 1 integron inside SGI1; ^i^: gene encoding beta-lactam resistance; ^j^:gene encoding sulphonamide resistance.

### Bacterial strains

A total of 538 isolates selected from 8,663 serotype Typhimurium isolates from the French Food Safety Agency (AFSSA, Maisons-Alfort, France) collection were analyzed. They were isolated between 1999 and 2009 in France and identified as *Salmonella enterica enterica *serotype Typhimurium according to the White-Kauffmann-Le Minor scheme by agglutination with O- and H-antigen specific sera (BioRad, Marnes-la-Coquette, France). The *Salmonella *isolates are sent on a voluntary basis through a network 150 veterinary or food analysis laboratories covering different French districts. Sampling was carried out firstly to remove duplicate strains and to select different sources of isolation and secondly on a random basis. The selected isolates can be considered representative of the total collection of the *Salmonella *network. Thus, for each year, at least one representative isolate from the three main sectors--animals, food or the environment (natural environment or ecosystem)--was tested. Within each sector, we then selected strains from various food-animal sources (poultry, swine and cattle) including primary production sites, livestock farms and raw materials from processing sites or from domestic or wild species. As described in Table 2, isolates were from samples of pigs (n = 61), poultry (n = 212), cattle (n = 67) and from other minor domestic or wild animal species (n = 51). The latter included strains from birds (n = 11), sheep (n = 9), horses (n = 6), goats (n = 5), snakes (n = 2) and rabbits (n = 2). We also investigated strains isolated from the environment (n = 23) and food products (n = 90), including ready-to-eat foods (n = 16), pork (n = 28), dairy products (n = 14), beef (n = 6), seafood (n = 5), egg products (n = 5) and vegetables (n = 3). Analyses were also conducted on a panel of few clinical human *Salmonella *Typhimurium isolates (n = 28) collected by the National Reference Centre for *Salmonella *(Institut Pasteur, Paris) and selected according to their various sources and PFGE genetic diversity.

**Table 2:** Genotype distribution according to isolation sources

	Food Animal sources								
				
Genotype	**No**.	Pigs	Poultry	Cattle	Other species^1^	Food products^2^	Human	Environment	Unknown
A1	1			1					
A2	3	1	1		1				
A3	3		2			1			
A4	1			1					
A5	145	4	84	12	17	16	4	5	3
A6	1		1						
A7	3		1	1		1			
A8	2				1	1			
A9	53	5	25	5	6	10	1		1

B1	6	1	1		2	1		1	
B2	19	1	10	1	2	4		1	
B3	9	1	1	2	3		2		
B4	1	1							
B5	2			1	1				
B6	210	39	60	38	12	38	11	12	
B7	3	1	1			1			
B8	8		1	2			5		
B9	6	1	2	1		1			1
B10	2		1			1			
B11	1		1						
B12	2			1		1			
B13	4		4						
B14	2			1	1				
B15	1				1				

C1	1	1							
C2	21	4	7		1	6	1	1	1
C3	1		1						
C4	10	1	5		1	1	2		
C5	1					1			
C6	5				2	2	1		
C7	2		2						
C8	7		1			4	1	1	

D	1							1	

E	1							1	

Total	538	61	212	67	51	90	28	23	6

^1^Birds (11), Sheep (N = 9), Horses (N = 6), Goats (N = 5), Snakes (2), Rabbits (2), Unknown (16).

^2 ^Cooked dishes (16), Pork (28), Diary products (14), Beef (6), Seafood (5), Egg products (5), Vegetables (3), Unknown (13).

A set of control strains was used to validate the STM GeneDisc^® ^array (Table 3). Reference strain LT2 was used as a positive control for testing SPI genetic markers (*ssaQ*, *mgtC*, *spi4-D *and *sopB *genes), and virulence plasmid pSLT (*spvC *gene). Typhimurium strain 08CEB5766SAL was used as a negative control for testing the *ssaQ*, *sopB *and *spvC *markers, whereas the 00-01041 strain kindly provided by the Federal Institute for Risk Assessment (BfR) in Berlin, Germany, was used as a negative template to test the *spi4_D *and *mgtC *markers. All these negative control strains had been tested previously using conventional PCR.

**Table 3:** Set of control strains

Strain	Source	DT104 16S- 23S spacer	*ssaQ*	*mgtC*	*spi4_D*	*sopB*	*spvC*	SGI1 left Junction	*intI1*	*bla*_TEM_	*sul1*
LT2		-	+	+	+	+	+	-	-	-	-
05CEB1571SAL	ANSES	+	+	+	+	+	+	+	+	-	-
07CEB5289SAL	ANSES	-	+	+	+	+	-	-	+	+	+
07CEB9150SAL	ANSES	+	+	+	+	+	-	-	-	+	-
01CEB12158	ANSES	-	+	+	+	+	-	-	-	-	-
08CEB5766SAL	ANSES	+	-	+	+	-	-	-	-	-	-
63.48	DTU Food	+	+	+	+	+	-	-	-	+	-
61.12	DTU Food	-	+	+	+	+	-	-	+	+	+
00-01041	BfR			-	-						

The specificity of the phage type DT104 marker targeting the 16S-23S rRNA intergenic spacer region was tested with 43 strains of different phage types: atypical DT146 (n = 1), DT120 (n = 10), DT135 (n = 1), DT99 (n = 1), DT8 (n = 2), DT193 (n = 4), DT30 (n = 2), DT12 (n = 2), DT4 variant (n = 1), U302 (n = 12), DT2 (n = 1), DT208 (n = 1), DT12a (n = 1), DT136 (n = 1), DT18 (n = 1), DT36 (n = 1), U311 (n = 1) and 59 strains of phage type DT104.

Phage-typing had already been performed either in the Laboratory of Gastrointestinal Pathogens at the Health Protection Agency (HPA, London, UK) or in the National Reference Centre on *Salmonella *at the Institut Pasteur (Paris, France). The presence of SGI1 was explored by targeting the left junction sequence and detecting integrase of class 1 integron gene (*intI1*) and a sulfonamide resistance determinant (*sul1*). The positive control strain used for these three markers was *S*. Typhimurium strain 05CEB1571SAL, a strain isolated from turkey and well-characterized by a European project. Positive results had already been detected for the left junction sequence, *intI1 *and *sul1 *genes. Finally, the study validated detection of the beta-lactam resistance gene (*bla*_TEM_) by testing ANSES and European collection strains 07CEB5289SAL of serotype Virchow, 07CEB9150SAL and 63.48 of serotype Paratyphi B var.Java, and 61.12 of serotype Isangii carrying respectively *bla*_TEM-1 _(penicillinase-producing), *bla*_TEM-52_, *bla*_TEM-20 _and *bla*_TEM-63 _variants linked to ESBL phenotypes (Table 3).

For test purposes, bacteria were cultured from a single colony on agar plates and grown overnight at 37°C. DNA from a small aliquot of the colony corresponding to approximately 2 × 10^6 ^bacteria was extracted using the InstaGene matrix (Bio-Rad Laboratories) and 36 μL of the DNA extracts were tested using the STM GeneDisc^® ^array.

### Data Analysis

Results are based on reaction curves and other features of real-time PCR that can be analyzed and printed as tables with MS Excel (Microsoft). To normalize results, a maximum cycle threshold--indicating the PCR cycle th at shows a significant increase in the fluorescence signal compared to the background--and minimum fluorescence amplitude were defined at 30 cycles and 500 arbitrary fluorescence units respectively. All percentage values for each genetic marker were calculated with their confidence interval at 95% according to a Fisher-Snedecor distribution. For phage-type DT104 determination, the specificity calculation was the proportion of negative tests which are true negative. The sensitivity was the proportion of positive tests which are true positive.

The normalized presence or absence of each gene determinant for each strain was analyzed as character values using BioNumerics software version 5.1 (Applied Maths, Sint-Martens-Latem, Belgium). A cluster analysis was performed with the Dice coefficient using the unweighted pair group method with arithmetic averages (UPGMA dendrogram). Cluster analysis was used to define different groups of genotypes, the term "genotype" indicating strains with a similar gene determinant profile.

## Results

### Prevalence of gene determinants in serotype Typhimurium strains

#### -Virulence determinants

All the investigated strains carried the *ttrC *marker specific to the *Salmonella *genus. The virulence potential of Typhimurium strains was characterized by testing five virulence-associated determinants. Four of them are located on SPI-2 to -5 and one, *spvC*, is related to the *Salmonella *Typhimurium virulence plasmid (pSLT). Each marker was tested against one positive strain (LT2) and against a specific negative control. The efficiency of each marker was checked and validated. SPI determinants are well conserved and usually present in all *Salmonella enterica *strains because they were acquired during *Salmonella *evolution [7]. Nevertheless, in this study, some atypical strains (n = 5) were observed and tested negative for one or two SPI markers.

We found three strains that were negative for *ssaQ*, and a single strain negative for *spi4_D *or *sopB*. These results suggest that there has been deletion or changes in the SPI-2 and/or SPI-4 region. The *mgtC *marker was detected in all 538 tested strains, indicating that the SPI-3 region was always present, whatever the strain (Table 4). The *spvC *gene pSLT determinant was frequently present (80 to 90%) in the studied strains whatever their isolation source (Table 4). These results are consistent with a recent virulotyping study on a large European collection of *Salmonella *strains, where *spvC *was found only in serotype Typhimurium (68%) and Enteritidis (85%) among the five serotypes regulated in Europe [12]. In the same study, SPI-1 to -5 determinants were conserved in the five serotypes.

**Table 4:** Distribution of gene determinant among isolation sources

		Percentage of gene determinant presence (confidence interval at 95%)									
		
Sources	No. of isolates	DT104 16S-23S spacer	*ssaQ*	*mgtC*	*spi4_D*	*sopB*	*spvC*	SGI1 left junction	*intI1*	*bla*_TEM_	*sul1*
Pigs	61	66 (52.31-77.27)	100 (95.21-100)	100 (95.21-100)	98 (91.2-99.96)	100 (95.21-100)	89 (77.78-95.26)	67 (54-78.69)	75 (62.71-85.54)	18 (9.36-29.98)	75 (62.71-85.54)
Poultry	212	34 (27.62-40.76)	100 (98.60-100)	100 (98.60-100)	100 (98.60-100)	100 (98.60-100)	80 (74.18-85.33)	37 (30.29-43.67)	39 (32.54-46.07)	10 (6.24-14-74)	41 (34.35-47.98)
Cattle	67	65 (53.06-76.85)	100 (95.63-100)	100 (95.63-100)	98 (91.96-99.96)	100 (95.63-100)	86 (76.03-93.67)	65 (53.06-76.85)	71 (59.31-81.99)	8 (2.47-16.56)	76 (64.14-85.69)
Other animal species^1^	51	31 (24.13-51.92)	98 (89.55-99.95)	100 (94.3-100)	98 (89.55-99.95)	100 (94.3-100)	82 (69.13-91.6)	31 (19.11-45.89)	43 (29.35-57.75)	12 (4.44-23.87)	47 (32.93-61.54)
Food products^2^	90	53 (42.51-63.93)	100 (96.73-100)	100 (96.73-100)	100 (96.73-100)	100 (96.73-100)	78 (67.79-85.87)	49 (38.2-59.65)	52 (41.43-62.87)	11 (5.46-19.49)	56 (44.7-66.04)
Human	28	71 (51.33-86.78)	100 (89.85-100)	100 (89.85-100)	100 (89.85-100)	100 (89.85-100)	86 (67.33-95.97)	57 (37.18-75.54)	64 (44.07-81.36)	36 (18.64-55.93)	64 (44.07-81.36)
Environment	23	61 (38.54-80.29)	96 (78.05-99.89)	100 (87.79-100)	100 (87.79-100)	96 (78.05-99.89)	91 (71.96-98.93)	61 (38.54-80.29)	61 (38.54-80.29)	9 (1.07-28.04)	65 (42.73-83.62)
Unknown	6	0 (0-39.3)	100 (60.7-100)	100 (60.7-100)	100 (60.7-100)	100 (60.7-100)	67 (22.28-95.67)	0 (0-39.3)	17 (0.42-64.12)	33 (4.22-77.72)	17 (0.42-64.12)

Total	538	47	99	100	99	99	82	47	52	12	54

#### -*Salmonella *genomic Island (SGI1) determinants

The SGI1 structure was detected using the left junction region, the integrase of class 1 integron gene (*intI1*) and the *sul1 *resistance determinant located in the multidrug resistance region. The left junction sequence, *intI1 *and *sul1 *genetic markers are all closely associated with SGI1. Not surprisingly, the frequencies of each marker were similar. Nevertheless, some strains carrying *intI1 *and/or *sul1 *were negative for the left junction region. Moreover, a few *sul1 *positive strains were negative for *intI1 *and/or the left junction region. Such results could suggest these markers are plasmid-mediated.

#### - Phage type DT104 determinant

The phage type DT104 determinant was explored by targeting a 16S-23S rRNA intergenic spacer sequence. This sequence is considered to be specific to DT104 strains [4]. Positive and negative control strains were used for this marker. Of the 59 confirmed DT104 strains, all but four were positive. Furthermore, the sequence was not detected in the atypical DT146 (n = 1), DT120 (n = 1), DT135 (n = 1), DT99 (n = 1), DT8 (n = 2), DT193 (n = 4), DT30 (n = 3), DT12 (n = 2), DT4 variant (n = 1), U302 (n = 12), DT2 (n = 1), DT208 (n = 1), DT12a (n = 1), DT18 (n = 1), DT36 (n = 1) or U311 (n = 1) strains. However, we observe a cross-reaction with one DT136 strain and nine of the ten DT120 strains investigated out of the 102 strains tested. The specificity and sensitivity values for this gene target were of 89.5% and 84.6% respectively. The DT104 marker was detected in 47% of the 538 tested strains with unequal distribution among isolate sources. This marker was carried by 71% of human strains (Table 4). Furthermore, the DT104 marker was observed in around 60% of environmental samples. Nearly half the food product strains carried this marker, while the lowest frequencies occurred in poultry and other animal species, with around 40% of positive strains.

#### - Antimicrobial resistance determinants

Beta-lactam resistance including ESBL and non-ESBL producing strains was explored by targeting a family of *bla*_TEM _genes encoding TEM beta-lactamase enzymes. Reference positive strains carrying *bla*_TEM-1_, *bla*_TEM-20_, *bla*_TEM-52 _and *bla*_TEM-63 _were correctly detected with the GeneDisc^® ^array.

The *bla*_TEM _determinant was unequally distributed among the tested strains. The highest level--36%--was detected in human isolates. In animal or food sources, it was found in around 10 to 20% of strains (Table 4).

Sulfonamide resistance was detected by targeting the *sul1 *determinant, most often associated with the SGI1 gene cluster and phage type DT104 strains. *sul1 *rates varied according to isolation sources, the highest levels being found in swine (75%) and bovine (74%) isolates and the lowest in poultry (41%) and other minor animal species (47%).

### Assignment of Typhimurium genotypes

**Figure 1:**
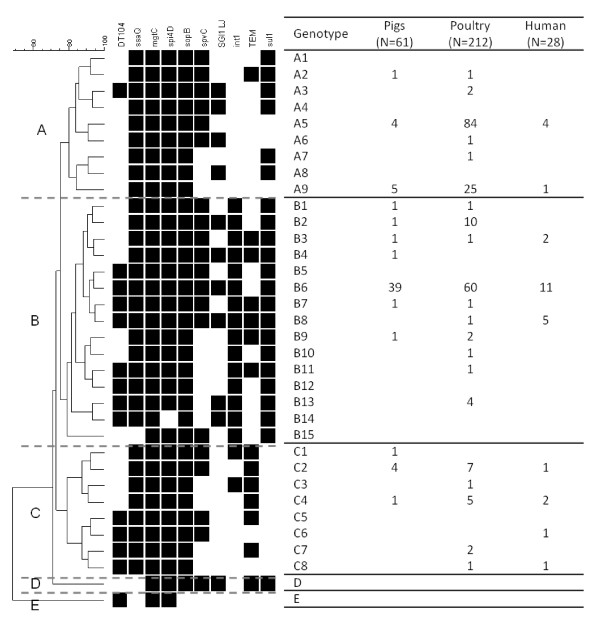
**Genotype constructed with the Unweighted Pair Group Method using arithmetic Averages (UPGMA) on total investigated strains with strain distribution in the main isolation sources: poultry, pigs and human sources**. A black box indicates the presence of the genotype's determinant gene. SGI1 LJ means "SGI1 Left Junction".

All the strains were classified according to their genotype determined by the combination of the ten investigated markers. Using this combination of markers, the 538 strains were grouped into 34 different genotypes according to the UPGMA method. A dendrogram was generated using the Dice correlation coefficient. Genotypes were clustered into three main groups and two minor groups named A to E (Figure 1 and Table 2).

Group A was composed of 211 strains divided into nine profiles: A1 to A9. Some of them were relatively rare, whereas two were very frequently observed (A5 and A9 genotypes). Strains in this group were usually negative for the DT104 determinant (98%) but positive for the sulfonamides resistance marker (*sul1 *gene). The class 1 integron marker (*intI1*) was never detected, though some Group A strains harbored the SGI1 determinant. Moreover, the beta-lactam resistance determinant TEM was present in three strains with A2 profiles. The major genotype A5 accounted for 67% of Group A strains and was linked to the presence of all four SPI determinants and the plasmid-associated *spvC *determinant. A second profile, A9, occurred more frequently than the others, accounting for 24% of Group A strains. A5 and A9 genotypes were very closely related as the A9 profile shared the A5 determinant profile, differing only by the absence of *spvC*. Both profiles were encountered every year in strains from various sources (Figure 1 and Table 2). Group B was the largest, containing 276 strains. The 15 genotypes of Group B were distributed throughout the 10-year study period (1999-2009). The most common genotype was B6, detected in all types of sources and encountered in 76% of Group B strains (n = 210). All determinants except the *bla*_TEM _gene were positive in this genotype. The other 14 profiles were much less frequent (Table 2). Furthermore, 84% of Group B strains were positive for the DT104 marker. Group B strains consistently exhibited *sul1 *and *intI1 *determinants, whereas 88% of these strains (n = 244) carried the SGI1 left junction marker. As previously reported, the SGI1 left junction region was not conserved among all isolates [8]. Atypical profiles were detected in three strains, of which two were isolated from rabbit farms and feces. These two strains were negative for the *spi_4D *determinant located on SPI-4 and assigned to the B14 profile. The third atypical strain, isolated from an eagle, was negative for the *ssaQ *marker and assigned to the B15 profile (Figure 1).

Group C included 49 strains divided into 8 genotypes that were found throughout the study period. All strains from Group C were negative for *sul1 *marker. They were also negative for *intI1 *and SGI1 left junction determinants except for two *intI1 *positive strains (C1 and C3 profiles) isolated either from poultry or swine sources. Likewise, the DT104 marker was rare, observed in only 6.5% (n = 15) of Group C strains (Figure 1).

Two other minor groups--D and E--were identified, each composed of a single strain. Genotypes derived from these groups were considered atypical and uncommon. Some SPI virulence genes were missing: *ssaQ *for the single Group D strain and both *mgtC *and *spi4D *for the Group E strain. Group D and E strains were both recovered from environmental samples, suggesting the presence of such atypical isolates in ecosystem niches (Figure 1 and Table 2).

### Association of the genetic markers from poultry, swine and human sources

Strains from human and swine sources showed the three SGI1-associated markers (*sul1*, *intI1 *and SGI1 left junction) more frequently (57.1% and 61.2% respectively) than those from poultry (35.4%). For the three markers, statistical differences in percentages were observed between swine and poultry sources. Table 4 highlights the finding that poultry-source strains harbored all the investigated determinants less frequently, with the exception of SPI-associated genes.

In poultry sources (Figure 1 and Table 2), great diversity was observed as 21 different genotypes were identified and distributed over the main three groups, A, B and C. Six different genotypes identified in Group A accounted for 54% of the isolates (n = 114 strains) mainly detected in two major genotypes A5 and A9. These two genotypes are those with low-marker patterns and account for more than half of the poultry strains. The frequently-encountered B6 and B2 genotypes were also detected for 33% of poultry strains out of a total of 10 different genotypes found in poultry sources The five Group C genotypes contained few poultry strains (n = 16) compared to the total. In swine sources, the 61 strains were assigned to 13 genotypes (Figure 1 and Table 2). Most of the strains were categorized in seven Group B genotypes, especially B6 (64%). A single strain of genotype C1 was detected in a swine source. All these Group B and C strains carried most of the tested determinants, especially the three SGI1-associated markers and the antimicrobial resistance determinants. Finally, the 28 strains from human sources were divided into nine different genotypes. The human strains shared the same genotypes as the poultry or swine strains whether in Group A, B or C, with the exception of a single strain that exhibited the C6 pattern never found in other sources. Sixty-four percent of Group B human strains carried the SGI1 determinant (64%). Genotype B8, positive for all determinants was almost distributed in human source (5 out 6 strains).

## Discussion

Over the past decade, serotype Typhimurium has been the most prevalent among *Salmonella enterica *subsp. *enterica *serotypes in human and animal sources worldwide. Furthermore, multiple-antibiotic-resistant strains have emerged, most often linked to phage type DT104. Many data regarding both the emergence and increase of phage type DT104 strains over the past years are available in some countries [13,14]. In contrast, no recent data are available regarding phage-type frequencies in French Typhimurium strains. A recent publication highlighted the lack of standardization of the phage-typing method within laboratories [15]. Detecting the phage type DT104 determinant using the GeneDisc^® ^appears to be a valuable fast alternative method for monitoring isolates. Markers for SGI1 (left junction region), DT104 (16S-23S intergenic spacer region) and antibiotic-resistance (*sul1*) were tested in the GeneDisc^® ^array developed here. Analysis of the results confirm the link between the three markers as previously described [16-18] and show that most of the tested Typhimurium collection carried all three markers simultaneously. Thus, of the 538 isolates tested, 210 (39%) were assigned to genotype B6, the most common genotype of the 34 identified. The B6 genotype was characterized by the presence of all ten tested markers, except the *bla*_TEM _gene. Other genotypes were closely related to B6, differing by only one or two markers. The majority of occurrences of B6 and B8 genotypes characterized by a high number of markers were host-specific. They have been observed in 64%, 60% and 57% of pig, cattle and human isolates respectively whereas only detected in 28% of poultry sources. The integrase of class 1 integron (*intI1*) is usually detected in isolates carrying SGI1. In our study, the *intI1 *determinant was only detected in 52% of the overall panel of isolates. In contrast, the two strains assigned to genotype B5 were positive for the DT104 marker and *intI1 *but negative for the SGI1 left junction and also exhibited a multi-drug-resistant phenotype. Another study also described this situation and concluded that class 1 integron gene cassettes should be detected in 48.5% of *Salmonella *isolates in which the SGI1 left junction is absent [8]. In another study, one DT104 strain [12] presented the same pattern associated with an ACSSuT pattern indicating the presence of an SGI1 variant in which molecular determinants could not be detected.

Our results revealed 36% *bla*_TEM_-positive strains in human strains and 11% in animal strains. Beta-lactamase production continues to be the leading cause of resistance to beta-lactam antibiotics among gram-negative bacteria. Furthermore, there have been reports of an increased incidence and prevalence of extended-spectrum beta-lactamases (ESBLs) in recent years. The first ESBLs arose in the early 1980 s from mutation from widespread, broad-spectrum beta-lactamases such as TEM-1 or SHV-1. Monitoring the frequency of *bla*_TEM _in *Salmonella *is therefore a major public health concern. In our study, we identified 14 different genotypes harboring the *bla*_TEM _gene, representing 13% of isolates (68 isolates). The most frequent *bla*_TEM _gene source was observed in human isolates (36%), whereas it was detected in only 8% of environment-source strains and 11% of animal and food-product isolates. These results are consistent with a study performed on French *Salmonella *Typhimurium isolates to determine *bla*_TEM _emergence in human and non-human sources which revealed the presence of *bla*_TEM _in 26% of human isolates and 23% of animal isolates [19,20]. Of the 14 different *bla*_TEM _genotypes, six of the Group B genotypes were always associated with the *intI1 *marker. The *intI1 *gene includes a site-specific recombination system capable of integrating and expressing genes contained in structures known as mobile gene cassettes. Integrons are described as a structure with high gene diversity in cassettes and a major reservoir of antibiotic-resistance genes, suggesting a broad role in adaptation during bacterial evolution and a major public health concern. In our study, the presence of *intI1 *from SGI1 in the absence of the SGI1 left junction was observed in nine Group B genotypes, two Group C genotypes and never in Group A. Moreover, all the Group B genotypes harboring the *bla*_TEM _gene contained the *sul1 *determinant. Other such atypical strains were encountered during a European study on the molecular sub-typing of *Salmonella *genomic islands on a large collection of isolates from different countries. This last study highlighted a correlation between *spvC *positive strains and the presence of *bla*_TEM _not observed in the current study [8]. One of the main genotypes, A9, exhibited the four SPI-2 to -5 determinants in the absence of all the other targeted genes. A frequent, closely-related A5 genotype also harbored the same SPI pattern in addition to the plasmid-associated *spvC *determinant. Along with the B6 and C2 genotypes, these two major A5 and A9 genotypes were detected in all sources, particularly human, poultry and swine sources, which suggest that they are widespread throughout various niches. *Salmonella *plasmid-encoded virulence factors are a selective advantage to some *Salmonella *variants for colonizing new niches over the course of *Salmonella *evolution [21]. Our finding also indicates that Typhimurium strains could share common combinations of markers whatever their source. In contrast, some genotypes were unique to animal sources: A3, A6, B10, B11, B13 and C3 were unique to poultry sources; B4 and C1 were unique to swine sources. No genotypes were assigned exclusively to human strains, but the number of clinical strains tested was fairly low. Although the studied collection of strains was representative of the main animal and food sources, the *Salmonella *network collects *Salmonella *isolates on a voluntary basis. There may, therefore, have been some bias in the selected strains, especially for serotype Typhimurium mainly serotyped in other veterinary or food analysis laboratories. Moreover, the number of strains tested from each source was not evenly distributed. The high proportion of poultry isolates is due to European regulations in this production sector, leading to many surveillance and sampling programs with monitoring and official controls.

Studies suggest that *Salmonella *plasmid-encoded virulence factors are a selective advantage to some *Salmonella *variants for colonizing new niches over the course of *Salmonella *evolution [21].

## Conclusion

The GeneDisc^® ^macroarray presented in this study made it possible to easily explore variability of the ten relevant gene determinants within Typhimurium very quickly during a on-hour run. Based on the presence or absence of these markers, 34 different marker combinations (genotypes) were observed among the 538 studied isolates, recovered mainly from food, animal or human sources. Three major genotypes were defined, being observed in 75% of the studied strains. Although SPI determinants were almost always present, our findings show variation in the detection of other gene determinants, especially for the SGI1 specific determinants (left junction and *intI1), sul1 *and *bla*_TEM_. In a microarray-based study on the characterization of *Salmonella *subspecies I isolates, most intra-serotype variation involved differences in only a few regions of the core genome [22]. This is the case for serotype Typhimurium. This study found major variation in the presence or absence of other gene determinants, as most of these determinants are plasmid- or transposon-mediated. These variations can be explained by intra-serotype horizontal gene exchanges that generate numerous genotype combinations. These horizontal gene transfer events may also occur between serotypes, as described in some studies demonstrating SGI1 lateral transfer from serotype Typhimurium to other serotypes [23,24]. This study highlighted variations in genotype frequencies according to source. Low-marker determinant genotypes were mostly detected in poultry sources, whereas high-marker determinant genotypes were observed in swine, cattle and human sources.

Serotyping cannot detect intra-serotype variation, so microarrays are currently most commonly used for comparative genome hybridization and gene expression studies. Nevertheless, although the high-density microarray-based approach has become more popular, these tools are limited by the availability of skilled personnel and require sophisticated equipment generally not available in routine surveillance laboratories [25,26]. This study demonstrates a very simple, specific, high-throughput, real-time multiplex PCR-based method that can determine genotypes for a preliminary analysis of Typhimurium intra-serotype diversity. Based on the same principle, the GeneDisc^® ^system can be enhanced and extended to other pertinent targets and genes according to the issue to be addressed, such as serotype identification or emerging new resistance mechanisms.

## Authors' contributions

The macro-array was designed by PF, MB and AB. MB performed all the laboratory analyses. The results were analyzed and interpreted by MB, PF and AB. SAG gave special attention to the antimicrobial resistance aspect of data and the choice of control strains. FXW was responsible for the clinical isolates and performed some phage-typing assays. All the authors were involved in drafting or revising the manuscript. The authors read and approved the final manuscript.
